# Signaling and metabolic properties of fast and slow smooth muscle types from mice

**DOI:** 10.1007/s00424-017-2096-6

**Published:** 2018-01-29

**Authors:** Lena Boberg, Ferenc L. M. Szekeres, Anders Arner

**Affiliations:** 1 Department of Physiology and Pharmacology, Karolinska Institutet, v Eulers v 8, 171 77 Stockholm, SE Sweden; 2 School of Health and Education, University of Skövde, Skövde, Sweden

**Keywords:** Contractile kinetics, Energy metabolism, Myosin isoforms, Phasic, Shortening velocity, Tonic

## Abstract

This study aims to improve the classification of smooth muscle types to better understand their normal and pathological functional phenotypes. Four different smooth muscle tissues (aorta, muscular arteries, intestine, urinary bladder) with a 5-fold difference in maximal shortening velocity were obtained from mice and classified according to expression of the inserted myosin heavy chain (SMHC-B). Western blotting and quantitative PCR analyses were used to determine 15 metabolic and 8 cell signaling key components in each tissue. The slow muscle type (aorta) with a 12 times lower SMHC-B had 6-fold lower expression of the phosphatase subunit MYPT1, a 7-fold higher expression of Rhokinase 1, and a 3-fold higher expression of the PKC target CPI17, compared to the faster (urinary bladder) smooth muscle. The slow muscle had higher expression of components involved in glucose uptake and glycolysis (type 1 glucose transporter, 3 times; hexokinase, 13 times) and in gluconeogenesis (phosphoenolpyruvate carboxykinase, 43 times), but lower expression of the metabolic sensing AMP-activated kinase, alpha 2 isoform (5 times). The slow type also had higher expression of enzymes involved in lipid metabolism (hormone-sensitive lipase, 10 times; lipoprotein lipase, 13 times; fatty acid synthase, 6 times; type 2 acetyl-coenzyme A carboxylase, 8 times). We present a refined division of smooth muscle into muscle types based on the analysis of contractile, metabolic, and signaling components. Slow compared to fast smooth muscle has a lower expression of the deactivating phosphatase and upregulated Ca^2+^ sensitizing pathways and is more adapted for sustained glucose and lipid metabolism.

## Introduction

Smooth muscle performs a wide range of important physiological tasks, including the rapid contractions of the urinary bladder to expel urine and the sustained tension maintenance in the wall of arteries to maintain blood pressure. Although they all share the common smooth structural appearance, the smooth muscle types are uniquely adapted to their requirements and exhibit a broad range of structural, mechanical, regulatory, and metabolic properties. Still, it has not been possible to clearly identify specific smooth muscle types. In early work by Emil Bozler [8], smooth muscle was divided in two main groups, “single unit” (unitary/visceral) and “multiunit” types, based on their regulation via motor nerves and cellular electrical coupling. Although this division is present in most textbooks on muscle physiology, it does not fully describe the diversity of the tissue. More recently, the group of Somlyo and Somlyo divided smooth muscles in “phasic” and “tonic” types, based on the contractile responses to activation [20, 49, 52]. The phasic muscles, e.g., in the mesenteric vein and ileum, exhibit a rapid, transient, contractile response, whereas the tonic muscles, e.g., in large arteries, have a slower force development, but a sustained contraction. It was subsequently shown that these two groups of muscle have differences in cellular signaling, contractile kinetics, and nucleotide affinity, e.g., [17, 18, 21, 22, 28]. In general, comparative data show a variation in several other properties of tonic and phasic smooth muscles, e.g., [16].

It seems logical to divide muscles according to the properties of contractile system, and in the striated muscle the division into muscle types can be made based on the expression of the myosin heavy chain genes, which correlates with the contractile kinetics [46]. In smooth muscle, myosin is generated from one main smooth muscle heavy chain gene with the addition of non-muscle myosins [53]. Still, smooth muscle exhibits a large span in contractile kinetics from slow arterial muscles to the almost seven times faster visceral types [36]. It has been shown that faster kinetics of actin–myosin interaction in smooth muscle correlates with a higher relative expression of an inserted myosin heavy chain isoform [27] and with a higher relative expression of a smooth muscle variant of the essential 17-kDa myosin light chain (LC17a, [36, 51]). The exact role of these smooth muscle myosin isoforms in determining the cross-bridge kinetics is still not fully clear, both potential mechanisms have been challenged [1, 27], and it is possible that these variants are expressed in a coordinated way. Additional considerations are, e.g., the role of the non-muscle myosin, which can be expressed in significant amounts in smooth muscle and support a slow contraction [32, 38, 43] and other regulatory proteins, e.g., calponin [23]. Irrespective of potential mechanisms, a strong correlation is observed between the maximal shortening velocity, reflecting cross-bridge turnover, and the expression of the myosin heavy chain insert and the essential light chain isoforms [3], both parameters being potential markers for the smooth muscle contractile phenotype.

The smooth muscle contractile system is, in comparison to that of striated muscles, more complex with regard to its regulation since the myosin-based phosphorylation process is subject to an extensive signaling network affecting the Ca^2+^ sensitivity of the contraction, mainly via the RhoA-Rhokinase and protein kinase C (PKC) pathways, e.g., [50]. Also, regarding this aspect of smooth muscle contraction, tissue variability has been reported in several studies, e.g., regarding phosphatase expression, and PKC and RhoA expression [18, 40, 54].

The contractile and regulatory processes in muscle are tightly linked to cellular energy turnover. The metabolism of intact smooth muscle tissue has been studied, and pioneering work demonstrated a low energy turnover and high lactate production under aerobic conditions, cf. Paul [41, 42]. A variation in metabolic tension cost between smooth muscles is seen, cf. [41, 42], but a more extensive link between smooth muscle type and metabolism is not available.

Smooth muscle thus converts metabolic energy to mechanical work regulated by an extensive and complex cellular signaling. It is possible that an improved division into smooth muscle types can be achieved by considering the three main components of the smooth muscle contraction: contractile properties, cellular signaling, and energy metabolism. Although the smooth muscle is multicellular and complex, such a consideration at the tissue level might be an important first step to understand how smooth muscle is adapted to its physiological environment and altered in response to pathological events and disease. The main objective of this study was therefore to develop a characterization of signaling and metabolism in relation to contractile kinetics by comparing different types of smooth muscle tissues from one species. We report on four smooth muscle tissues from mouse, with a large span in contractile kinetics, where we measured 15 metabolic and 8 cell signaling components, and summarize the main characteristics of fast and slow smooth muscle phenotypes.

## Methods

### Animals and tissues

The study was performed on adult female and male C57/Bl6 mice. The animals were euthanized by cervical dislocation and the different smooth muscle tissues were dissected. We included urinary bladder (freed from urothelium), muscular arteries (superior mesenteric artery and femoral artery), aorta, and the outer longitudinal layer of the small intestine. The tissue samples (about 1–2 mg) were rapidly frozen in liquid N_2_ and kept at − 80 °C until analysis with gel electrophoresis or real-time PCR. All husbandry and experiments were approved by the local animal ethics committee and conformed to the European Convention for the Protection of Vertebrate Animals used for Experimental and other Scientific Purposes (Council of Europe No. 123, Strasbourg 1985).

### PCR analysis of mRNA expression

The mRNA was extracted (RNeasy Kit; Qiagen) from four different types of smooth muscle tissue (urinary bladder, intestine, aorta, femoral artery). Quality and concentrations were assayed using a NanoDrop spectrophotometer at 260/280 nm (> 1.9) and 260/230 nm (> 2.0). High Capacity Reverse Transcription Kit (Applied Biosystems) was used to generate cDNA. The cDNA of the metabolic enzymes was analyzed (in duplicates) by quantitative real-time PCR (RT-qPCR) using 96-well plates on an Applied Biosystems real-time PCR using Fast SYBR Green MasterMix (Applied Biosystems). All samples were diluted to the same concentration before running the qPCR. Primers for each gene are listed in Table 1. We confirmed using agarose gel electrophoresis that all primers resulted in one PCR product. The amount was quantified using the relative standard curve method with the pooled samples from all tissues as a standard and normalized to HPRT (hypoxanthine–guanine phosphoribosyltransferase).

**Table 1:** Primers used for RT-qPCR analysis of the mouse samples

Protein, NCBI mRNA accession number	Forward	Reverse
ACC2 NM_133904	GAATCTCACGCGCCTACTATC	CCTGCACAGAGATTTCACCGT
AMPKα1 NM_001013367	GTCAAAGCCGACCCAATGATA	CGTACACGCAAATAATAGGGGTT
AMPKα2 NM_023991	AAGATCGGACACTACGTCCTG	TGCCACTTTATGGCCTGTCAA
FAS NM_007988	GGAGGTGGTGATAGCCGGTAT	CTGGGCTCTATGGATTACCCA
G6PDH NM_173371	ATGAAGCACACAGGCATTTGG	GGACTGTTTCAGCTATACCTGGA
GLUT1 NM_011400	TCAAACATGGAACCACCGCTA	TTCCTTCTCTGTCGGCCTCTT
GLUT4 NM_009204	TCCCTTCAGTTTGGCTATAACATTG	TGAACAGAGCTACAATGCAACGT
HEXOK2 NM_013820	GTGTGCTCCGAGTAAGGGTG	CCACATTGCCGAATGCCTG
HPRT NM_013556	GCAGTACAGCCCCAAAATGG	TTGGATACAGGCCAGACTTTGTT
HSL NM_001039507	CACCCATAGTCAAGAACCCCTTC	CGGTGACGCTGAAAGTGGTAGA
LDHA NM_001136069	CATTGTCAAGTACAGTCCACAC	TCCCAAAAACCGAGTAATTGGAA
LPL NM_008509	AGGGCTCTGCCTGAGTTGTA	ACCAGGCCTTCGAAATTTCT
MCD NM_019966	CTGTCGCCTATCCCTGGATTC	AGAAATCTCAGCCGTTACCGG
PEPCK NM_011044	ATCTTTGGTGGCCGTAGACCT	AAATACCTGGCCCACTGGC
Pyruvate kinase NM_001253883	GGCTGAATTTCTCTCATGGAACC	CTACCGTCCTGTTGCGGTG
TFAM NM_009360	GGAATGTGGAGCGTGCTAAAA	TATTCCGAAGTGTTTTTCCAGCA

All primers except AMPKα1 and AMPKα2 were obtained from [48]

*ACC2* acetyl-coenzyme A carboxylase beta; *AMPKα1* AMP-activated, alpha 1 catalytic subunit; *AMPKα2* AMP-activated, alpha 2 catalytic subunit; *FAS* fatty acid synthase; *G6PDH* hexose-6-phosphate dehydrogenase (glucose 1-dehydrogenase); *GLUT1* solute carrier family 2 (facilitated glucose transporter); *GLUT4* solute carrier family 2 (facilitated glucose transporter); *HEXOK2* hexokinase 2; *HPRT* hypoxanthine–guanine phosphoribosyltransferase; *HSL* lipase, hormone sensitive; *LDHA* lactate dehydrogenase A; *LPL* lipoprotein lipase; *MCD* malonyl-CoA decarboxylase; *PEPCK* phosphoenolpyruvate carboxykinase 1; *TFAM* transcription factor A, mitochondrial

Standard PCR (40 cycles), agarose gel electrophoresis (4% gels), and analysis using Quantity One software (Bio-Rad) were used for the determination of the relative concentration of inserted smooth muscle myosin heavy chain (SMHC-B) using the following primers [33]: forward, ATGTACAAGGGCAAGAAGAGGC; reverse, GAGGAGTTGTCGTTCTTGAC. The inserted and non-inserted forms gave two distinct bands (330 and 351 bp), and the relative content of the larger product of the inserted mRNA was evaluated.

### Western blot analysis

The tissue expression of signaling components (in urinary bladder, intestine, aorta, and superior mesenteric artery) was analyzed essentially as described previously [7]. In principle, the samples were pulverized using a glass micromortar, dissolved in homogenizing buffer, 1% SDS, 1 mM Na_3_VO_4_, 1% phenylmethylsulfonyl fluoride (PMSF), centrifuged, and analyzed for protein content using the Bio-Rad Protein assay (Bio-Rad, Richmond, CA, USA). The samples were analyzed by SDS-PAGE using a Bio-Rad MiniGel system (Bio-Rad, Richmond, CA). Equal amounts of protein from each sample were loaded on the polyacrylamide gels. After blotting onto nitrocellulose membranes, the bands were visualized using primary antibodies, the enhanced chemiluminescence kit (ECL, Amersham Bioscience), and analyzed using Quantity One software (Bio-Rad). The respective antibodies and polyacrylamide concentration were RhoA (dilution 1:1000, Santa Cruz, 12%), ROCK1 (1:500, Santa Cruz, 7%), ROCK2 (1:500, Santa Cruz, 7%), MYPT-1 (1:500, Santa Cruz, 7%), CPI-17 (1:1000, Upstate, 15%), PKCα (1 : 500, Santa Cruz, 12%), Rho GDI (1 : 500, Santa Cruz, 15%), and PP 1 β (2.5 μ g /mL, Calbiochem, 12%).

### Statistics

All values are given as mean ± SEM, with the number of samples in parentheses. Statistical comparisons were made using Student’s *t* test or, and when appropriate, analysis of variance and the Holm–Sidak method for multiple comparisons (*p* < 0.05 was considered significant) using log values for the qPCR and Western blot data. Calculations and curve fitting were performed using SigmaPlot and SigmaStat for Windows (SPSS Science, Chicago, IL).

## Results

### Myosin heavy chain isoform expression

As a basis for our comparisons between smooth muscle tissues, with different contractile kinetics, we determined the relative content of mRNA for the inserted myosin heavy chain (SMHC-B). The results are shown in Table 2. The lowest expression was observed in the slow arterial muscles (aorta and the muscular artery) and the highest in the fast muscle type (urinary bladder). The intestinal ileum muscle had intermediate expression, with about 25% lower relative content than in the urinary bladder. For the presentation of the signaling and metabolic components below, we plotted the data against the relative expression of the inserted heavy chain isoform (SMHC-B), as a marker for the contractile kinetics, with low and high contents indicating slow and fast muscles, respectively.

**Table 2:** Relative expression of the inserted smooth muscle myosin heavy chain (SMHC-B) mRNA in different smooth muscle tissues of the mouse (*n* = 6)

	Aorta	Mesenteric artery	Intestine	Urinary bladder
SMHC-B/total SMHC	0.072 ± 0.005	0.111 ± 0.006	0.590 ± 0.019	0.905 ± 0.005

### Expression of signaling components

**Fig. 1:**
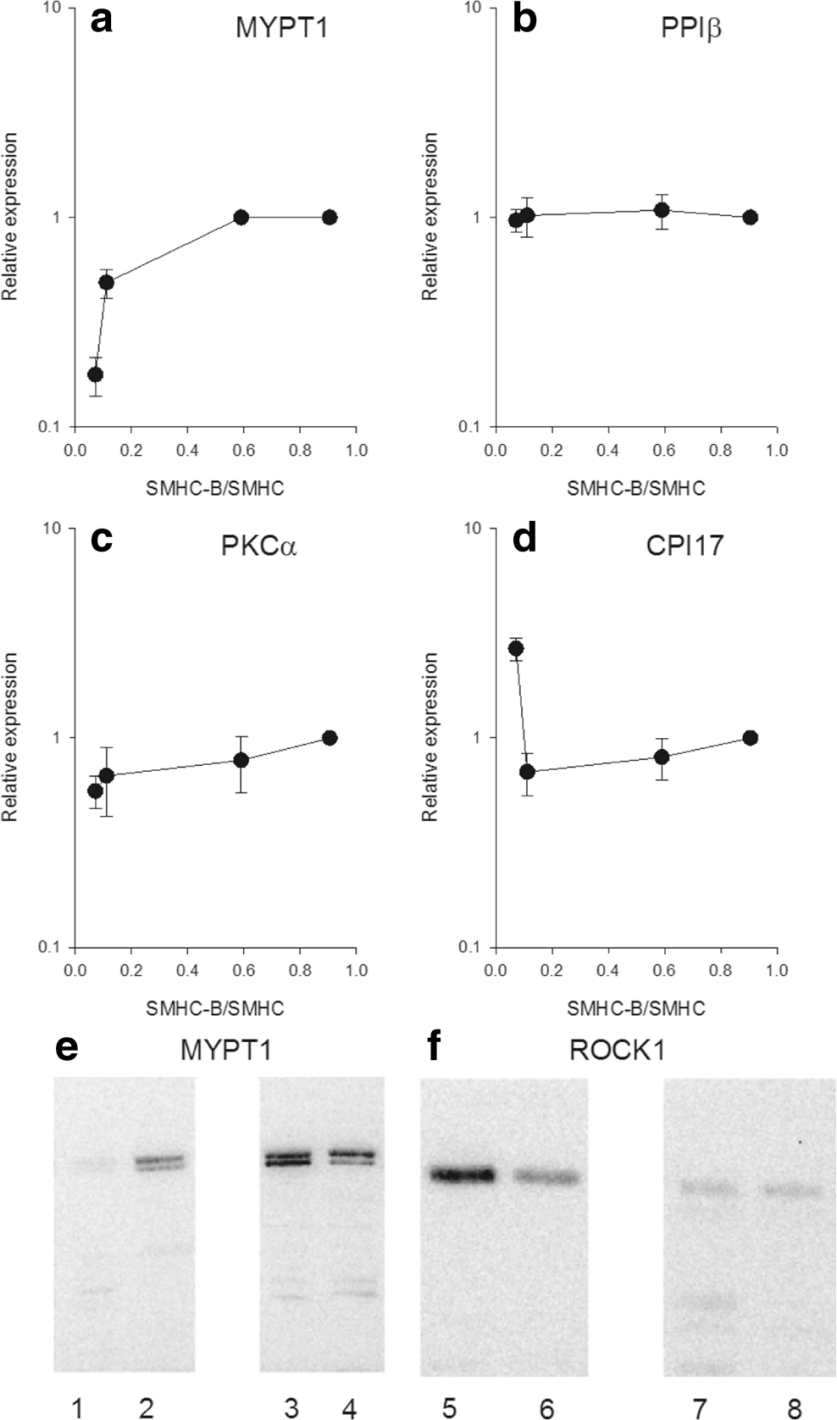
Expression of signaling components in smooth muscles with different relative expression of SMHC-B. The data show from left to right: aorta, mesenteric artery, ileum, and urinary bladder, *n* = 4–8. The Western blot data for the signaling components are given relative to the expression in the urinary bladder. **a** MYPT1, **b** PP1β, **c** PKCα, **d** CPI17. **a** Aorta *p* < 0.05 compared to all other groups, femoral artery *p* < 0.05 compared to ileum. **b**, **c** No significant differences. **d** Aorta *p* < 0.05 compared to all other groups. Panels (**e**) and (**f**) show Western blots for MYPT1 and ROCK1, respectively, from two separate gels. Equal amounts of protein from aorta (*lanes 1*, *5*), mesenteric artery (*lanes 2*, *6*), intestine (*lanes 3*, *7*), and urinary bladder (*lanes 4*, *8*) were separated. The third lane on each gel (white area) contained another sample not examined in this study

**Fig. 2:**
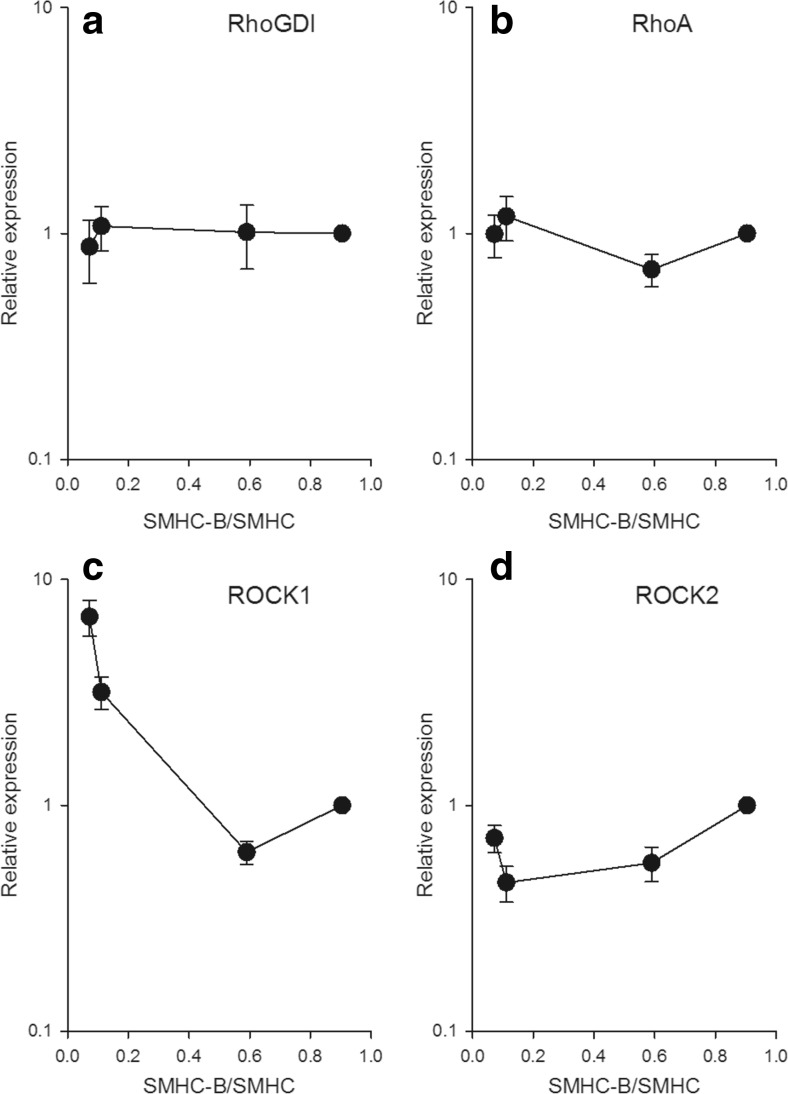
Expression of signaling components in smooth muscles with different relative expression of SMHC-B. The Western blot data show from left to right: aorta, mesenteric artery, ileum, and urinary bladder, *n* = 3–8. The data for the signaling components are given relative to the expression in the urinary bladder. **a** RhoGDI, **b** RhoA, **c** ROCK1, **d** ROCK2. **a**, **b**, **d** No significant differences. **c** Aorta *p* < 0.05 compared to all other groups, mesenteric artery *p* < 0.05 compared to ileum and urinary bladder

A first step was to examine the expression of selected components in the contractile activation, including the myosin light chain phosphatase and the two main Ca^2+^-sensitizing pathways (Rho-Rhokinase and PKC). For this purpose, we used Western blotting and antibodies previously used for characterization of cell signaling in mouse urinary bladder [7]. For each signaling protein, the amount was normalized to the expression in the fast smooth muscle tissue (i.e., urinary bladder). The content of the myosin targeting subunit (MYPT1) of the myosin light chain phosphatase increased with increasing relative SMHC-B expression, with an about six times higher amount (measured as the total amount including two isoforms separated in the gel) in the fast ileum and urinary bladder compared to the slow aorta smooth muscle (Fig. 1a and Western blot in Fig. 1e), whereas the catalytic subunit, PPIβ (Fig. 1b), did not differ between tissues. For the protein kinase C (PKC) pathway, the PKCα content (Fig. 1c) was similar in the tissues. The CPI17 (Fig. 1d) was highly expressed in the aorta, with the other tissues exhibiting a lower and similar expression. For the Rho-Rhokinase Ca^2+^-sensitizing pathway, the RhoGDI (Fig. 2a), RhoA (Fig. 2b), and ROCK2 (Fig. 2d) did not correlate with the relative SMHC-B expression, whereas the ROCK1 (Fig. 2c and Western blot in Fig. 1e) showed a correlation with SMHC-B expression, slower muscles having higher content.

### Expression of metabolic components

Our next step was to determine expression of components in the energy metabolism pathways in relation to contractile kinetics. This profiling included a larger number of proteins, previously not analyzed in detail in smooth muscle, and we therefore used a RT-qPCR approach, with primers previously used in the mouse ([48], Table 1). We used the femoral artery as an example of a muscular artery. We selected key proteins in the main pathways of energy metabolism (cf. Table 1): (1) *glucose uptake/glycolysis* (the glucose transporters GLUT 1 and GLUT 4, hexokinase II, and pyruvate kinase), (2) *gluconeogenesis* (PEPCK = phosphoenolpyruvate carboxykinase), (3) *lactate production* (LDH = lactate dehydrogenase A), (4) *the pentose phosphate pathway* (G6PDH = hexose-6-phosphate dehydrogenase), (5) *mitochondrial turnover* (TFAM = mitochondrial transcription factor A), (6) *the metabolic sensor AMP-kinase* (AMPKα1 and α2 = AMP-activated, alpha 1 and 2 catalytic subunits), and (7) *the lipid hydrolysis and synthesis* (HSL = hormone-sensitive lipase, LPL = lipoprotein lipase, FAS = fatty acid synthase, MCD = malonyl-CoA decarboxylase, ACC2 = acetyl-coenzyme A carboxylase beta). For each enzyme, we examined the mRNA expression in relation to the myosin heavy chain expression (SMHC-B) and identified the tissues with significantly higher or lower expression.

**Fig. 3:**
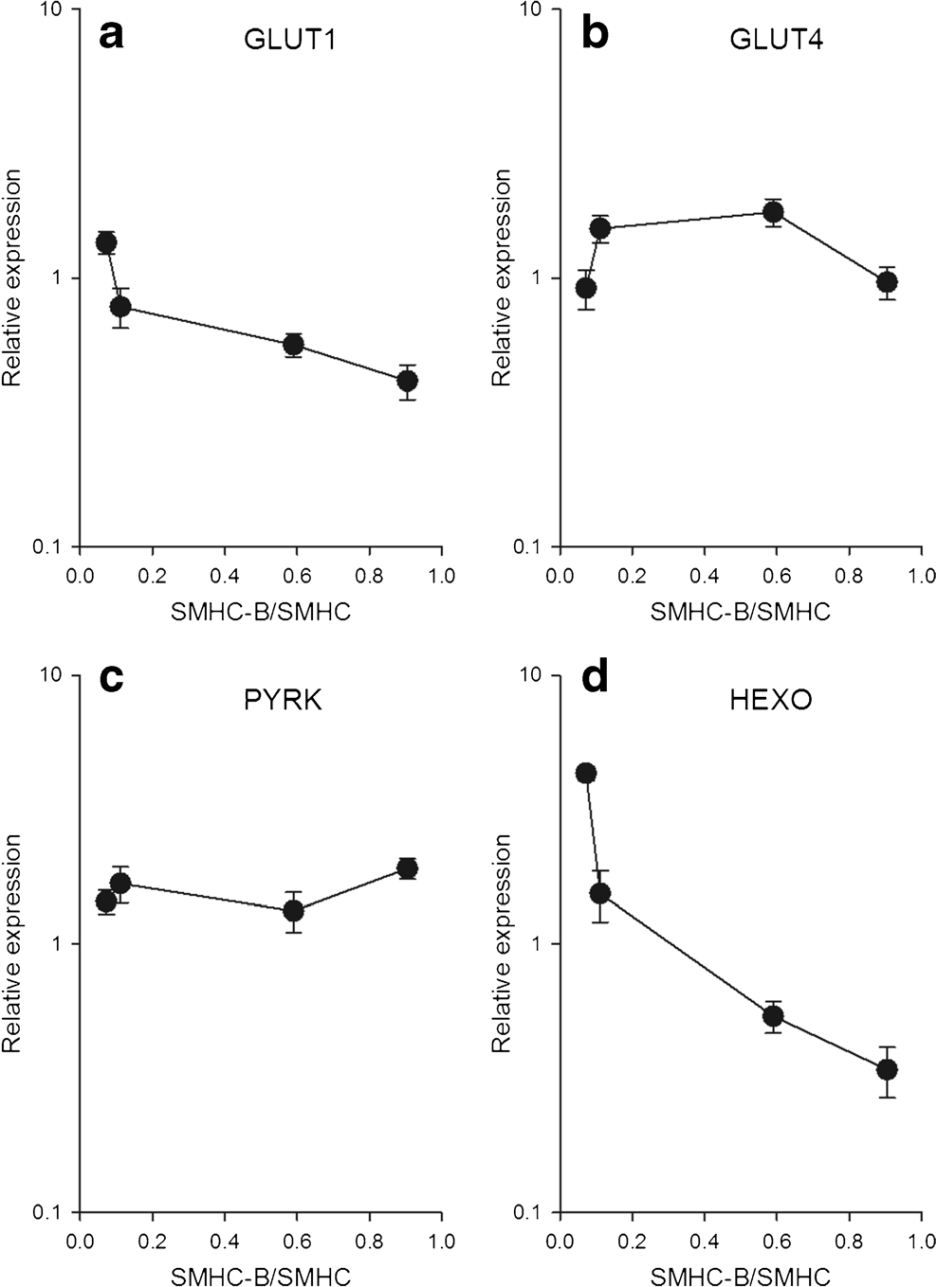
Expression of metabolic components in smooth muscles with different relative expression of SMHC-B. The RT-qPCR data show from left to right: aorta, femoral artery, ileum, and urinary bladder, *n* = 9–10. **a** GLUT1, **b** GLUT4, **c** PYRK, **d** HEXO. **a** Aorta *p* < 0.05 compared to all other groups, femoral artery *p* < 0.05 compared to urinary bladder. **b** Ileum *p* < 0.05 compared to bladder and aorta, femoral artery *p* < 0.05 compared to aorta. **c** No significant differences. **d** All groups are significantly (*p* < 0.05) different

**Fig. 4:**
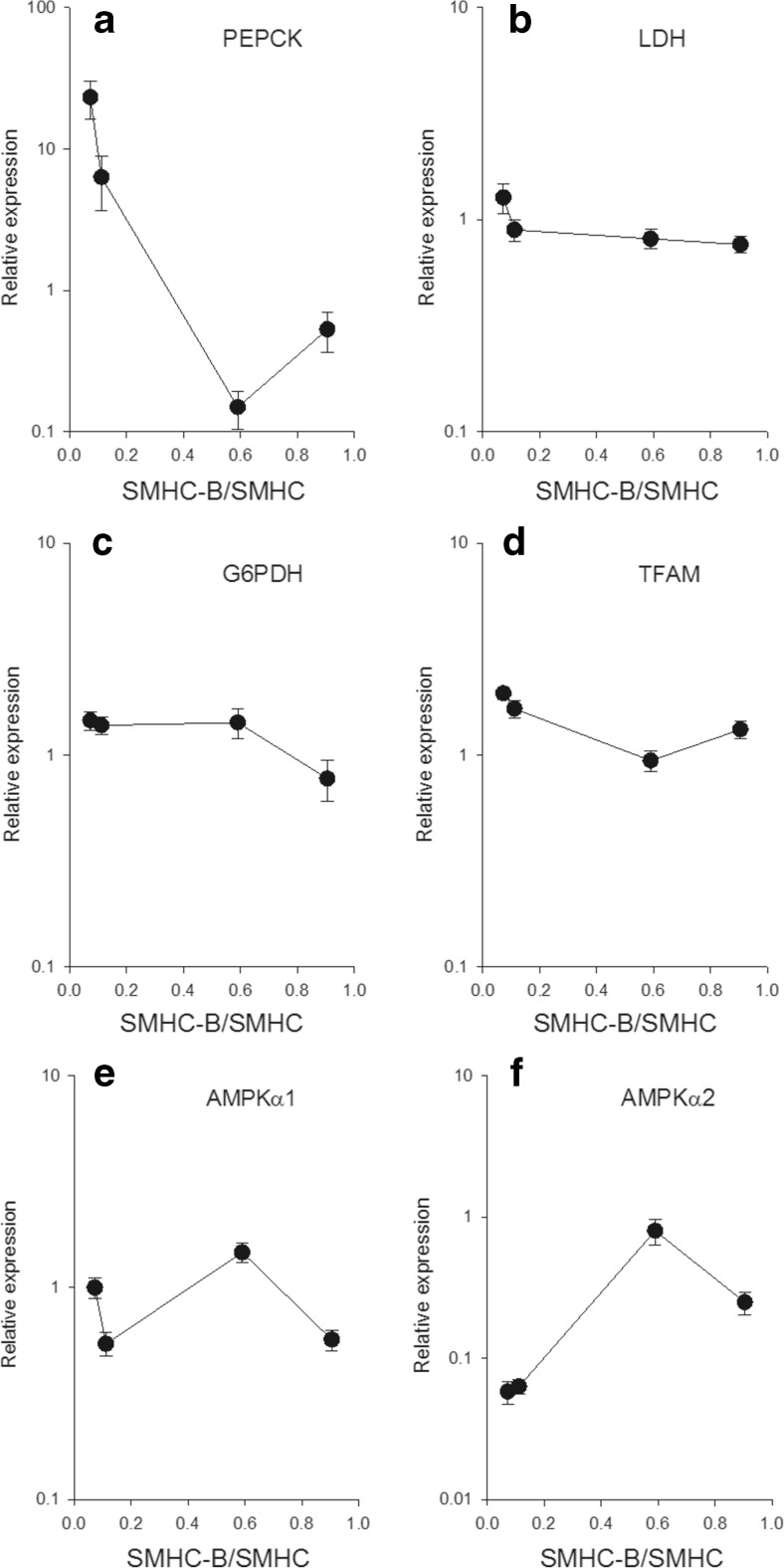
Expression of metabolic components in smooth muscles with different relative expression of SMHC-B. The RT-qPCR data show from left to right: aorta, femoral artery, ileum, and urinary bladder, *n* = 9–10. **a** PEPCK, **b** LDH, **c** G6PHD, **d** TFAM, **e** AMPKα1, **f** AMPKα2. **a** Aorta *p* < 0.05 compared to all other groups, femoral artery *p* < 0.05 from ileum and urinary bladder. **b** No significant differences. **c** Urinary bladder *p* < 0.05 compared to all other groups. **d** Ileum *p* < 0.05 compared to all other groups, aorta *p* < 0.05 compared to urinary bladder. **e** Ileum *p* < 0.05 compared to femoral artery and urinary bladder, aorta *p* < 0.05 compared to femoral artery and urinary bladder. **f** Ileum *p* < 0.05 compared to all other groups, urinary bladder *p* < 0.05 compared to aorta and femoral artery

**Fig. 5:**
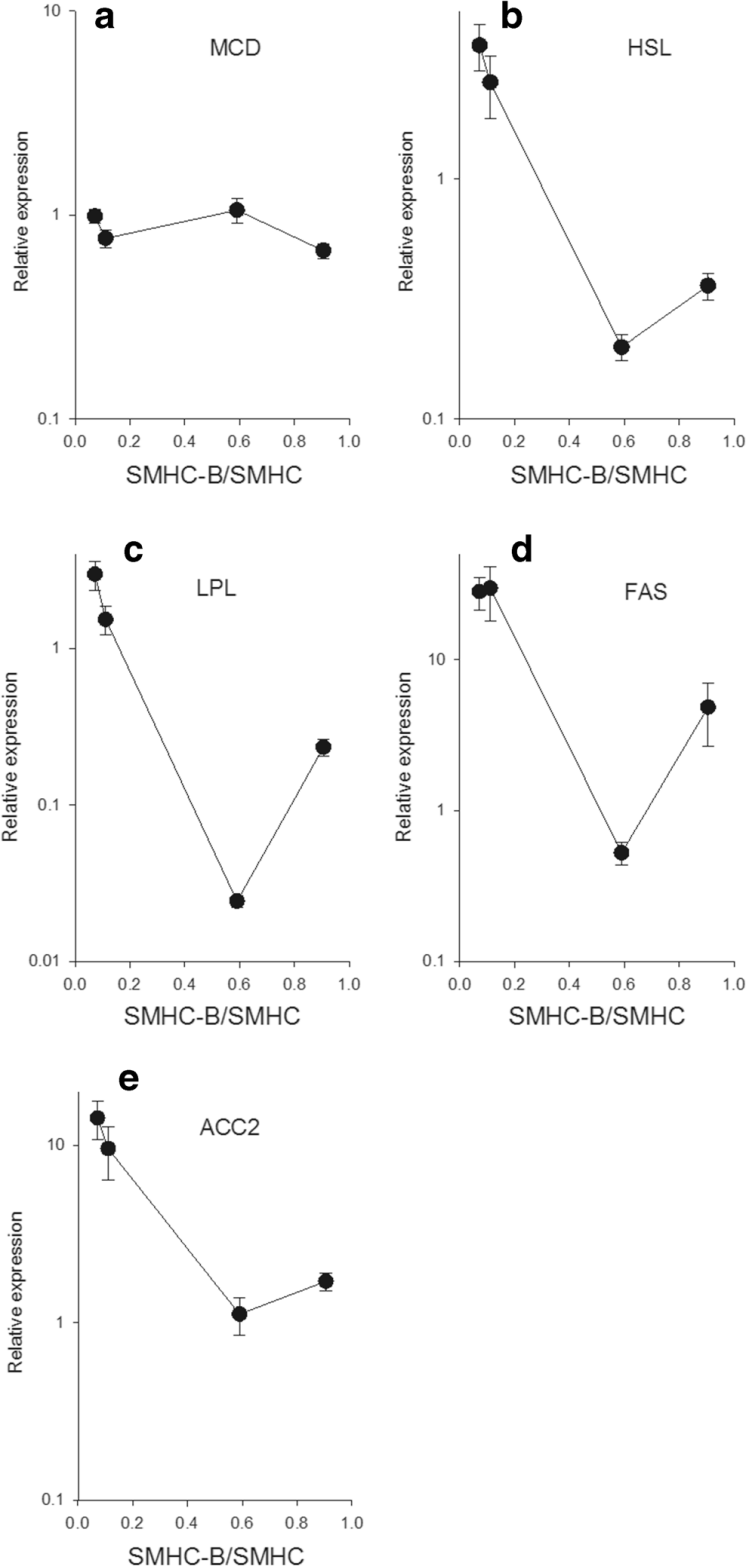
Expression of metabolic components in smooth muscles with different relative expression of SMHC-B. The RT-qPCR data show from left to right: aorta, femoral artery, ileum, and urinary bladder, *n* = 9–10. **a** MCD, **b** HSL, **c** LPL, **d** FAS, **e** ACC2. **a** Urinary bladder *p* < 0.05 compared to aorta and ileum. **b** Aorta and femoral artery *p* < 0.05 compared to ileum and urinary bladder. **c** All group significantly (*p* < 0.05) different. **d** Aorta and femoral artery *p* < 0.05 compared to ileum and urinary bladder, ileum *p* < 0.05 compared to urinary bladder. **e** Aorta and femoral artery *p* < 0.05 compared to ileum and urinary bladder

For the glycolysis pathway, both the glucose transporter GLUT4 (Fig. 3b) and the pyruvate kinase (PYRK, Fig. 3c) were expressed, with similar expression and no clear correlation with relative SMHC-B expression. However, the GLUT 1 transporter (Fig. 3a) and the key enzyme for glucose uptake, hexokinase (Fig. 3d), were negatively correlated with relative SMHC-B expression. The PEPCK (Fig. 4a) was highly expressed in the slow muscles and negatively correlated with relative SMHC-B expression. The LDH (Fig. 4b), G6PDH (Fig. 4c), TFAM (Fig. 4d), and the AMP-kinase α1 isoform (Fig. 4e) had similar expression and no clear correlation with relative SMHC-B expression. The AMP-kinase α2 isoform (Fig. 4f) was higher in the faster muscles. No clear correlation with SMHC-B expression was noted in the MCD (Fig. 5a) between tissues. The expression of the two lipolysis enzymes, HSL and LPL (Fig. 5b, c), and of the FAS and ACC2 (Fig. 5d, e) were lower in the faster muscles with the higher SMHC-B expression.

## Discussion

This study is an initial approach to develop a systematic view on the diversity of smooth muscle contractile system, including a consideration of contractile, signaling, and metabolic properties. We base our analysis on four smooth muscle tissues with a large span in contractile properties, obtained from one species, the mouse. The main idea is to correlate the mechanical diversity to expression of signaling and metabolic pathways, and Table 3 summarizes the main properties of slow and fast smooth muscles.

**Table 3:** Comparison of key contractile, signaling, and metabolic parameters of fast and slow smooth muscle types

Parameters	“FAST”	“SLOW”
Contractile parameters		
Representative tissues	Urinary bladder	Aorta
Shortening kinetics	High *V*_max_	~ 5 times slower [34, 36]
Tension development	High rate of tension development	~ 2 times slower [36]
Myosin types	High LC17a High SMHC-B Low non-muscle myosin	~ 16 times lower [3, 36] ~ 12 times lower Higher non-muscle myosin [3, 32]
ADP dependence	Low MgADP affinity	~ 36 times higher affinity [34]
ATP dependence	High MgATP affinity	~ 4 times lower affinity [34]
Phosphate dependence	High P_i_ inhibition of force	~ 2–4 times lower P_i_ inhibition of force [34]
Cell signaling		
Dephosphorylation of myosin	High expression of targeting subunit, MYPT1 no difference in light chain phosphatase, catalytic subunit PP1β	~ 6 times lower No difference
Protein kinase C pathway	Low CPI17 PKCα no difference	~ 3 times higher No difference
Rho-Rhokinase pathway	Low ROCK1 No difference in ROCK2, RhoA, and RhoGDI	~ 7 times higher No difference
Metabolic parameters		
Glucose uptake and glycolysis	Lower insulin-independent GLUT1 No difference in insulin-dependent GLUT4 Low hexokinase PYRK no difference	~ 3 times higher No difference ~ 13 times higher No difference
Lactate production	LDH no difference Low LDH-H	No difference ~ 1.4 times higher [37]
NADPH synthesis	G6DPH no difference	No difference
Gluconeogenesis	Low PEPCK	~ 43 times higher
Mitochondrial signaling	TFAM no difference	No difference
Metabolic sensing	AMPKα1 no difference High AMPKα2	No difference ~ 5 times lower
Lipid metabolism	Low HSL Low LPL	~ 10 times higher ~ 13 times higher
Lipid synthesis	Low FAS Low ACC2 MCD no difference	~ 6 times higher ~ 8 times higher No difference

To give an indication of the magnitude in differences, relative changes are approximated. Data obtained in the present study (estimated from mRNA or protein expression) are presented, and when appropriate, literature references are given (including other smooth muscle tissues with fast and slow characteristics)

We included a large elastic artery (aorta), muscular arteries (mesenteric artery and femoral artery), intestinal smooth muscle (longitudinal muscle of ileum), and the urinary bladder (detrusor muscle). The following values for the maximal shortening velocity (*V*_max_, in muscle lengths per second, ML/s, at 20–22 °C) in the different tissues can be obtained from the literature: (1) *aorta* (0.015–0.07 ML/s in guinea pig and mouse [34, 43], (2) *muscular artery* (mouse caudal artery ~ 0.08 ML/s at 37 °C [13], corresponding to about 0.02 at 22 °C using *Q*_10_ of 2.2 from [24]), (3) *intestine* (mouse ileum at 37 °C 0.29 ML/s [44], corresponding to 0.09 ML/s at 22 °C; guinea pig ileum and taenia coli 0.11–0.20 ML/s [33, 34, 36]), and (4) *urinary bladder* (mouse 0.19 ML/s [47]). The examined muscles in the present study seem to be divided into slow arterial (aorta, muscular artery) and fast visceral (bladder) muscle types. The intestine possibly represents an intermediary group, although it in many of the measured parameters resemble the fast urinary bladder.

We thus included muscles with an about 5-fold difference in *V*_max_ and showed, consistently with previous work, that the contractile kinetics (as evaluated from *V*_max_) correlates with expression of myosin heavy chain isoforms, cf. [3]. In general, *V*_max_ is also related to the rate of tension development, when different smooth muscles are compared [22, 36], suggesting that the kinetics of force generating cross-bridge transitions also vary between smooth muscle tissue types, although to a lower extent than *V*_max_ [36]. The tissues examined in the present study were obtained from mouse, but the difference in shortening velocity and myosin expression are also present in other experimental animals [3]. The slow arterial muscles could be denoted “tonic” and the fast visceral muscles “phasic” consistently with the classification of Somlyo and Somlyo [49], but could not readily be classified into “single” and “multiunit” types according to the Bozler nomenclature [8]. These earlier classifications are based on the contractile responses of intact muscles after activation and on the properties of the nervous control. We, however, use a division into “fast” and “slow” based on the contractile kinetics and myosin heavy chain isoform expression. The urinary bladder and the aorta would represent these extremes. We cannot on the basis of the current data set exclude a continuous distribution or the presence of well-defined intermediate groups.

The maximal shortening velocity (*V*_max_) in different muscles is generally correlated with the actin-activated myosin ATPase [6] and with the tension cost (i.e., the ATP turnover related to active force [11]). In this context, the generally slow and economical smooth muscle provides a physiological advantage over striated muscles during tension maintenance, e.g., in sustaining vascular tone [41]. Also, within the smooth muscle group, slower smooth muscles have a lower tension cost than comparatively faster smooth muscle types, e.g., rat aorta versus portal vein [2]. Characterizing the substrate and product dependence of cross-bridge interaction would give information on the rate-limiting reactions, and previous studies have compared slow and fast smooth muscle faster types [34]. In the slow muscle, *V*_max_ was less affected by lowering of [MgATP] and force was less inhibited by inorganic phosphate. However, the *V*_max_ of slow muscle is highly sensitive to MgADP [34] which correlates with a higher MgADP affinity to the rigor complex in slow tonic smooth muscle [17]. A further stronger MgADP binding is observed in smooth muscle expressing non-muscle myosin [32]. An important factor for determining the slow shortening velocity and tension economy is thus a high MgADP affinity, slowing cross-bridge detachment, reducing the shortening velocity, and increasing the duty cycle in slow smooth muscle. Although smooth muscles have an economic contraction, and would not “fatigue” and lose force during sustained contractions, in a similar manner as striated muscles, the high MgADP sensitivity, rather than P_i_ effects, will introduce an alternative mode of metabolic control of contraction affecting the contractile kinetics (*V*_max_). The pronounced effects of MgADP will introduce a strong dependence of contractile kinetics on energy metabolism.

The contractile kinetics of smooth muscle is tightly linked to the regulation of contraction. An important phenomenon is the “latch” state where dephosphorylation of cross-bridges introduces a slow cycling and tension maintenance [14]. Work from the Somlyo group [21] has demonstrated that tonic/slow smooth muscles have lower phosphatase activity compared to phasic/fast smooth muscles. Lower content of the targeting subunit of myosin light chain phosphatase (MYPT1) has been demonstrated in tonic versus phasic smooth muscle [54]. We show that the MYPT1 content is lower in the slow smooth muscle compared to the fast, but that the catalytic subunit (PPIβ) does not vary. Studies of newborn and hypertrophying urinary bladder smooth muscle have shown a lower expression of MYPT1 correlating with increased Ca^2+^ sensitivity [7, 15]. This suggests that the slow smooth muscle types exhibiting lower MYPT1 have a slower kinetics in the dephosphorylation reaction and a higher Ca^2+^ sensitivity compared to the faster smooth muscles. The latch phenomenon, i.e., the attachment of dephosphorylated myosin cross-bridges, is prominent in slow smooth muscles [14]. One would expect that a lower phosphatase activity would lead to a lower tendency for dephosphorylated attached (latch) cross-bridges. This suggests that factors other than the phosphatase activity per se will contribute to the increased probability for latch in slow muscle. It is possible that the slower turnover and increased time in attached cross-bridge states in slow smooth muscles will increase the probability for dephosphorylation and latch states, a process that would be further enhanced by metabolic generation of MgADP.

We examined the two main upstream modulators of the phosphatase in the regulation of smooth muscle, protein kinase C (PKC), and Rho-Rhokinase [50]. Although the PKCα expression showed little variation between the examined tissues, its target, the CPI17, was markedly higher in the slow tonic aorta, which correlates with previous data and the significant responses to PKC activation in this tissue [54]. RhoA and its modulator RhoGDI did not vary, but a clear correlation between SMHC-B and Rho-kinase 1 (ROCK1) expression was observed; the slower arterial muscles had higher ROCK1. This, in combination with a lower MYPT1 expression, might correlate to a difference in receptor activated Ca^2+^-sensitizing pathways and could result in a higher inhibition by Rho-kinase inhibitors in slow arterial versus fast bladder smooth**.**

In view of metabolic effects via MgADP on the cross-bridge kinetics, we addressed the question whether the difference in cross-bridge kinetics between smooth muscles could be linked to differences in metabolic energy turnover. Previous studies on smooth muscle have characterized the energy turnover associated with contraction, cf. [41, 42], and our study was primarily focused on differences in expression of metabolic pathways between smooth muscle types.

The GLUT1 and 4 glucose transporters were both expressed in the examined smooth muscles. Insulin receptors have been shown in smooth muscle [29], but the function of the insulin-responsive glucose transporter GLUT4 is less clear and the insulin-dependent glucose uptake is small [4]. Other studies have, however, shown expression of GLUT4 in smooth muscle, insulin-dependent glucose uptake, and effects on contractile function by the GLUT4 [e.g., 29, 9, 5, 39]. We show that the GLUT4 did not exhibit a correlation with SMHC-B expression, suggesting that the insulin-dependent glucose uptake is not a major factor determining the metabolic diversity. However, the insulin-independent GLUT1 was higher in slower muscles. Previous studies have shown that GLUT1 is expressed in aorta smooth muscle and that higher expression correlates with increased glucose uptake [25]. Hexokinase, the first and rate-limiting step in glycolysis, was markedly higher expressed in slow arterial muscles. Although the slow smooth muscles have lower energy consumption, they might require a continuous supply of energy to support more sustained contractions. The high expression of insulin-independent GLUT1 and of hexokinase would also make the slow arterial muscles more affected by high extracellular glucose levels, a potential factor of importance for development of atherosclerotic changes in large arteries in diabetic hyperglycemia.

Although the oxygen consumption in slow (aorta) smooth muscle is lower than in fast (portal vein) smooth muscle, the relative lactate production is higher in the slow muscle [2], reflecting an increased lactate production under aerobic conditions [41, 42]. The high expression of factors involved in glucose uptake in slow smooth muscle might be involved in providing glucose for ATP via glycolytic pathways producing lactate. The relatively higher lactate production is, however, not linked to altered expression of the lactate-producing M-isoform of lactate dehydrogenase. Indeed, the slow aorta instead expresses more of the H-form, less prone to produce lactate [37], suggesting that the conversion of pyruvate to lactate is not rate limiting for the lactate production in the slow muscle. No clear relationship could be demonstrated between pyruvate kinase (PYRK) and SMHC-B expression for all examined tissues. Since PYRK is the final step in glycolysis, an altered activity would not be a factor directing the glucose metabolism toward lactate in the slower smooth muscle. The aerobic glycolysis with lactate production is a prominent feature in smooth muscle [41, 42], similar to the Warburg effect of tumor tissues. The latter phenomenon has been linked to upregulation of hexokinase II [45], and the high expression of hexokinase can be an important contributor to the higher lactate production in slow smooth muscle. The glycolytic pathway has been proposed to support the activity of the membrane Na/K ATPase [35]. Interestingly, the lactate production decreases in the aorta when extracellular [Ca^2+^] and force are increased in depolarized muscle, whereas it increases in the portal vein [2], suggesting differences in regulation and recruitment of the aerobic glycolysis between different smooth muscle types. Although the slower muscles have higher expression of enzymes in glucose uptake and glycolysis, they also have markedly higher expression of PEPCK involved in gluconeogenesis, and the exact function of the varied PEPCK expression remains to be investigated.

Glucose-6-phosphate dehydrogenase (G6PDH) is a key enzyme in the pentose phosphate pathway, important for generating substrates for synthesis of nucleotides and producing NADPH which is involved in, e.g., fatty acid synthesis and protection against oxidative stress. G6PDH was slightly lower in the fast urinary bladder smooth muscle. The functional correlate to this finding is not yet clear. However, it has been shown that G6PDH is upregulated in isolated smooth muscle cells during mechanical/oxidative stress and that downregulation of the enzyme leads to increased oxidative stress [30]. In intact smooth muscle tissues, the activity of G6PDH is negatively correlated with the expression of contractile proteins [10], and a generally lower expression of G6PDH in the bladder might be important for maintaining a contractile phenotype. At the same time, oxidative stress of bladder smooth muscle has been proposed to be a pathological mechanism in bladder disorders [31], and a lower expression of G6PDH might be involved in these processes.

Adenosine monophosphate-activated kinase (AMPK) is a key metabolic sensor, activated by energy depletion and high AMP levels [19]. It affects several cellular functions including glucose uptake in skeletal muscle and inhibits agonist-induced contractions of arterial smooth muscle via effects on protein kinase C signaling [12]. Our profiling of AMPKα1 expression did not reveal any major differences between the examined tissues, although the AMPKα2 was slightly higher in the fast muscles, possibly reflecting higher demands on the metabolic control. TFAM, a key regulator of mitochondrial DNA [26], showed a small variation between the smooth muscle tissues with slightly higher values in the slow types, which could be a marker for a shift toward an oxidative metabolism possibly linked to a higher oxidation of fatty acids.

Both LPL and HSL enzymes responsible for uptake and breakdown of triglycerides were higher in the slower tonic muscles compare to the faster. This would suggest that the slower muscles are more adapted to metabolism of lipids. Although the MCD, important for fatty acid synthesis, was similar between tissues, both ACC2 and FAS, two enzymes involved in fatty acid synthesis, were highly expressed in slower muscles. This even further points the fatty acids as an important substrate for the metabolism in slower muscles.

In summary, the main purpose of this study was neither to examine the exact role of all metabolic and signaling proteins nor to examine all steps in the different pathways. Rather, we aimed at obtaining a “finger print” of cellular components as a basis for characterizing the “fast” and “slow” smooth muscle types. Further work based on key parameters identified in the present study (as outlined in Table 3), including a broader range of smooth muscle tissues, would enable a further insight into the variability of smooth muscle and possibly identification of intermediate types or improved pathological markers. In a physiological situation, a slow smooth muscle is found in large and muscular arteries, where it will stay contracted for longer times at low energy expenditure. This is achieved by expression of specific myosin isoforms, accompanied by slower rates of deactivation and lower phosphatase expression. The Rho-kinase 1 Ca^2+^-sensitizing component is highly expressed and in addition the sensitivity to PKC activation is enhanced. Although the energy turnover is low, the sustained contractions, while maintaining blood pressure, can be affected by varied levels of ADP resulting in “latch like” situations, which provides a link between contraction and a continuous input of metabolic energy from glucose and lipids.
